# Role of Proprotein Convertase Subtilisin/Kexin Type 9 in the Pathogenesis of Graves’ Orbitopathy in Orbital Fibroblasts

**DOI:** 10.3389/fendo.2020.607144

**Published:** 2021-01-08

**Authors:** Ga Eun Lee, Jinjoo Kim, Jihei Sara Lee, JaeSang Ko, Eun Jig Lee, Jin Sook Yoon

**Affiliations:** ^1^ Yonsei University College of Medicine, Seoul, South Korea; ^2^ Department of Ophthalmology, Severance Hospital, Institute of Vision Research, Yonsei University College of Medicine, Seoul, South Korea; ^3^ Department of Endocrinology, Severance Hospital, Yonsei University College of Medicine, Seoul, South Korea

**Keywords:** adipogenesis, Graves’ orbitopathy, inflammation, oxidative stress, proprotein convertase subtilisin/kexin type 9, PCSK9, thyroid eye disease

## Abstract

**Background:**

The proprotein convertase subtilisin/kexin type 9 (PCSK9) has been implicated in the pathogenesis of inflammatory diseases. We sought to investigate the role of PCSK9 in the pathogenesis of Graves’ orbitopathy (GO) and whether it may be a legitimate target for treatment.

**Methods:**

The *PCSK9* was compared between GO (n=11) and normal subjects (n=7) in orbital tissue explants using quantitative real-time PCR, and in cultured interleukin-1β (IL-1β)-treated fibroblasts using western blot. Western blot was used to identify the effects of PCSK9 inhibition on IL-1β-induced pro-inflammatory cytokines production and signaling molecules expression as well as levels of adipogenic markers and oxidative stress-related proteins. Adipogenic differentiation was identified using Oil Red O staining. The plasma PCSK9 concentrations were compared between patients with GO (n=44) and healthy subjects (n=26) by ELISA.

**Results:**

The *PCSK9* transcript level was higher in GO tissues. The depletion of PCSK9 blunted IL-1β-induced expression of intercellular adhesion molecule 1 (ICAM-1), IL-6, IL-8, and cyclooxygenase-2 (COX-2) in GO and non-GO fibroblasts. The levels of activated nuclear factor kappa-light-chain-enhancer of activated B cells (NF-κB) and phosphorylated forms of Akt and p38 were diminished when PCSK9 was suppressed in GO fibroblasts. Decreases in lipid droplets and attenuated levels of peroxisome proliferator-activated receptor gamma (PPARγ), CCAAT/enhancer-binding protein β (C/EBPβ), and leptin as well as hypoxia-inducible factor 1α (HIF-1α), manganese superoxide dismutase (MnSOD), thioredoxin (Trx), and heme oxygenase-1 (HO-1) were noted when PCSK9 was suppressed during adipocyte differentiation. The plasma PCSK9 level was significantly higher in GO patients and correlated with level of thyrotropin binding inhibitory immunoglobulin (TBII) and the clinical activity score (CAS).

**Conclusions:**

PCSK9 plays a significant role in GO. The PCSK9 inhibition attenuated the pro-inflammatory cytokines production, oxidative stress, and fibroblast differentiation into adipocytes. PCSK9 may serve as a therapeutic target and biomarker for GO.

## Introduction

Graves’ orbitopathy (GO) is an inflammatory autoimmune disorder, and it is the most frequent extrathyroidal manifestation of Graves’ disease (1). Clinical features of GO include upper eyelid retraction, edema and erythema of the periorbital tissue and conjunctiva, proptosis, corneal ulceration, and optic neuropathy. Three cell types which predominantly contribute to the development and progression of GO are B cells, T cells, and orbital fibroblasts (2). Stimulated by interactions with T cells and autoantibodies produced by B cells, orbital fibroblasts play a key role in the establishment of inflammation by producing cytokines, chemokines, and lipid mediators. Furthermore, they proliferate, synthesize extracellular matrix, and differentiate into adipocytes, leading to tissue remodeling characteristic of GO. The mainstay treatment for moderate-to-severe GO is systemic glucocorticoids therapy (3). Due to inadequate responses and adverse effects to glucocorticoids, however, there have been several investigations for other possible biological therapies (3).

The proprotein convertase subtilisin/kexin type 9 (PCSK9), which was first reported in 2003, is the ninth member of the protein convertase family (4). PCSK9 targets low density lipoprotein receptors (LDLR) on the hepatic cell surface, toward lysosomes for degradation, resulting in elevated serum LDL cholesterol levels (5). Now, PCSK9 inhibitors have emerged as novel therapeutics to treat cardiovascular diseases (6). However, current data suggest that PCSK9 inhibitors may have pleiotropic effects, affecting targets beyond LDLR (7–9). According to other studies, PCSK9 may be a key molecule in the pathophysiology of diseases such as atherosclerosis, myocardial ischemia, Alzheimer’s disease, psoriasis, and fatty liver disease (10–14). Studies on the PCSK9 in atherosclerosis, a chronic inflammatory disorder of vessel walls, showed that PCSK9 inhibition suppressed inflammatory cytokines production and decreased the activity of nuclear factor kappa-light-chain-enhancer of activated B cells (NF-κB) and intracellular cell adhesion molecule 1 (ICAM-1). Additionally, silencing PCSK9 decreased oxidative stress, apoptosis, proliferative capacity, and accumulation of macrophages (10, 14, 15). Numerous studies reporting the benefit of PCSK9 suppression *in vivo* and *in vitro* suggest that PCSK9 may be an attractive target in chronic inflammatory disorders (16). However, no previous studies have reported the effect of PCSK9 inhibition in GO.

In light of what is said above, this study was designed to investigate the role of PCSK9 in the pathogenesis of GO. We used small interfering RNA (siRNA) to promote cleavage of intracellular PCSK9 mRNA in orbital fibroblasts obtained from GO and normal subjects. We tested whether PCSK9 siRNA counteracts inflammation, proliferation, and adipocyte differentiation in orbital fibroblasts, the main pathogenic mechanisms in GO. In addition, we examined whether the plasma PCSK9 levels reflect the presence and the activity of GO using the clinical activity score (CAS).

## Materials and Methods

### Reagents

The antibodies were purchased from Santa Cruz Biotechnology (Santa Cruz, CA, USA), Cell Signaling Technology (Beverly, MA, USA), Novus Biologicals (Centennial, CO, USA), and Abcam (Cambridge, UK). The antibodies used in the study are listed in detail in **Supplementary Table 1**. PCSK9 siRNA and control siRNA were obtained from Santa Cruz Biotechnology, Inc. (Dallas, TX, USA). TransIT-siQUEST siRNA Transfection reagent was purchased from Mirus Bio, Inc. (Madison, WI, USA). The 3-(4,5-dimethyl-thiazol-2-yl)-2,5-diphenyl-tetrazolium bromide (MTT) assay and Oil Red O were products from Sigma-Aldrich, Inc. (Merck KGaA, Darmstadt, Germany). Dulbecco’s modified Eagle’s medium (DMEM), fetal bovine serum (FBS), penicillin, and gentamicin were purchased from Hyclone Laboratories, Inc. (Logan, UT, USA). Recombinant human interleukin-1β (IL-1β) and the enzyme-linked immunosorbent assay (ELISA) kit for PCSK9 were obtained from R&D Systems (Minneapolis, MN, USA).

### Subjects and Preparation of Tissues and Cells

Orbital tissue specimens were collected from GO subjects during orbital decompression surgery (nine females and two males; age 38–54 years). Non-GO orbital tissue was obtained during the course of upper (n=3) and lower lid (n=4) blepharoplasty from patients without history or clinical evidence of any thyroid disease (5 females and 2 males; age 36–57 years). Out of 11 GO and seven non-GO tissues, three GO and three non-GO tissues were randomly chosen for primary orbital fibroblast cultures. For gene expression analysis, nine out of 11 GO tissues were randomly selected, while all seven non-GO tissues were used. The study protocol was approved by the Institutional Review Board of Severance Hospital, and all participants provided written informed consent. This research adhered to the tenets of the Declaration of Helsinki. At the time of surgery, all GO patients were in euthyroid state and had not been administered steroid or radiation therapy for at least three months.

For plasma PCSK9 evaluation, 70 subjects were recruited: 22 with active GO (15 females and 7 males; age 42.41 ± 17.91 years), 22 with inactive GO (16 females and 6 males; age 40.64 ± 16.91 years), and 26 healthy volunteers (23 females and three males; age 36.69 ± 14.20 years). GO was considered “active” based on CAS, a grading system based on the seven classic features of inflammation in GO (17). Out of seven, GO was considered “active” if the CAS was ≥3. **Table 1** shows the demographic, clinical, and serologic data of the subjects.

**Table 1:** Clinical and serological characteristics of patient population for ELISA.

	Active GO (n=22)	Inactive GO (n=22)	Non-GO (n=26)	p-value
Sex (male/female)	7/15	6/16	3/23	0.213
Age (years), *M ± SD*	42.41 ± 17.91	40.64 ± 16.91	36.69 ± 14.20	0.575
Smokers, n (%)	6 (27.27)	4 (18.18)	2 (7.69)	0.202
PCSK9 (ng/ml)	256.46 ± 53.49	223.48 ± 36.42	190.83 ± 28.77	<0.001
CAS	4.45 ± 1.47	1.36 ± 0.66	—	<0.001
Duration GD (months), median (IQR)	8.55 (2–16)	7.18 (2–22)	—	0.396
T3 (0.58–1.59 ng/dl), *M ± SD*	1.17 ± 0.25	1.17 ± 0.26	—	0.796
Free T4 (0.70–1.48 ng/dl), *M ± SD*	1.19 ± 0.22	1.23 ± 0.21	—	0.751
TSH (0.35–4.94 μIU/ml), *M ± SD*	1.40 ± 0.84	1.36 ± 1.00	—	0.605
TBII (0–1.75 IU/L), *M ± SD*	18.09 ± 12.26	12.99 ± 7.42	—	0.231

ELISA, enzyme-linked immunosorbent assay; GO, Graves’ orbitopathy; SD, standard deviation; PCSK9, proprotein convertase subtilisin/kexin type 9; CAS, clinical activity score; GD, Graves’ disease; IQR, interquartile ranges; T3, triiodothyronine; T4, thyroxine; TSH, thyrotropin; TBII, thyrotropin-binding inhibitory immunoglobulin.

Orbital fibroblasts were isolated from the harvested tissue and cultured as described previously (18). After being minced, the tissue was placed directly in DMEM/F12 (in 1:1 ratio) medium containing 20% FBS, penicillin (100 U/ml), and gentamycin (20 μg/ml). Following incubation, tissues were maintained in solution containing DMEM, antibiotics, and 10% FBS. Once the growth of the fibroblasts was confirmed, the cells were treated with trypsin/ethylenediaminetetraacetic acid and passaged in monolayers. Strains were stored in liquid nitrogen and only those between the third and fifth passages were used for experiments.

### Cell Viability Assay

Cell viability was assessed with an MTT assay, following the manufacturer’s protocol (Sigma-Aldrich, Inc.). Orbital fibroblasts obtained from GO patients were seeded into 24-well culture plates (1 × 10^5^ cells/well) and treated with PCSK9 and control siRNAs (50 nM) for 10, 24, and 48 h. Thereafter, cells were washed and incubated in MTT solution (5 mg/ml) for 3 h at 37°C. Dimethyl sulfoxide (DMSO) was applied for solubilization and the absorbance of the converted dye was measured with a microplate reader (EL 340 Bio Kinetics Reader; Bio-Tek Instruments, Winooski, VT, USA) at 540 nm, with background subtraction at 630 nm.

### Quantitative Real-Time PCR

The RNA was extracted from cells using TriZol (Invitrogen, Carlsbad, CA, USA). Out of the extract, 1 μg was reverse-transcribed into cDNA (Qiagen, Valencia, CA, USA) and amplified with SYBR green real-time PCR master mix in a StepOne Plus real-time PCR thermocycler (Applied Biosystems, Foster City, CA, USA). The sequence of primers is listed in **Supplementary Table 2**. The PCR results for each type of mRNA were normalized to the level of GAPDH, and expressed as fold-change in the Ct value relative to the control group using the 2^-ΔΔCt^ method (19).

### Western Blot Assay

Equal amounts of protein (50 μg) were separated by 10% SDS polyacrylamide gel electrophoresis. The resolved proteins were transferred to nitrocellulose membranes and incubated overnight with primary antibodies at 4°C. Then, the membranes were probed with horseradish peroxidase–conjugated secondary antibodies. The bands were detected on X-ray films (GE Healthcare, Piscataway, NJ), and their intensities were quantified and normalized to that of the β-actin in the same sample.

### Adipogenesis

Using a previously published protocol, adipocyte differentiation of GO fibroblasts was induced (20). Cells were cultured in serum-free DMEM supplemented with T3, insulin (Boehringer-Mannheim, Mannheim, Germany), carbaprostaglandin (cPGI2; Calbiochem, La Jolla, CA, USA), and dexamethasone, along with proliferator-activated receptor gamma (PPARγ) agonist, rosiglitazone (10 μM; Cayman, Ann Arbor, MI, USA) for 7 days. To evaluate the effect of PCSK9 siRNA on adipogenesis, cells were transfected with PCSK9 siRNA for the entire 7-day differentiation period according to the manufacturer’s instructions.

### Oil Red O Staining

Cells were stained with Oil Red O as described by Green and Kehinde (21). A working solution was prepared by diluting 6 ml of a stock solution (0.5% Oil red O in isopropanol) with 4 ml of distilled water. The cells were fixed with 3.7% formalin at 4°C for 1 h before being washed with PBS and mixed with Oil Red O solution for 1 h at room temperature. The cell-solution mixture was visualized under on a light microscope (Olympus BX60; Olympus Corp., Melville, NY, USA).

### Blood Sampling and Measurement of Plasma PCSK9 and TBII Concentrations

Blood samples were drawn into test tubes containing sodium citrate. Platelet-free plasma was obtained after centrifugation at 1,500 g for 15 min at 4°C and stored at −80°C until analysis. Plasma PCSK9 levels were determined with a commercially available ELISA kit. All samples were tested in triplicate, and all sera were run in the same assay. The average value of three repeated assays was used for statistical analyses. Thyrotropin (TSH) binding inhibitory immunoglobulin (TBII) was measured with a third-generation TBII assay using the automated Cobas electrochemiluminescence immunoassay (Elecsys; Roche Diagnostics GmbH, Penzberg, Germany).

### Statistical Analysis

All experiments were performed in duplicate or triplicate on samples from each patient, and the results were expressed as mean ± standard deviation (SD). Comparisons of data between groups were performed with the independent *t*-test or ANOVA. The Bonferroni test was performed as a *post hoc* test. The Mann–Whitney *U*-test and Kruskal–Wallis test were used for nonparametric or not normally distributed data. Spearman’s rank correlation coefficient was used to analyze the correlation of plasma PCSK9 concentrations with CAS and plasma TBII levels. The SPSS for Windows, version 20.0 (SPSS, Inc., Chicago, IL, USA) was used. A *p*-value < 0.05 denoted statistical significance.

## Results

### GO Tissues Show Increased Expression of PCSK9, LDLR, and HIF-1α

**Figure 1:**
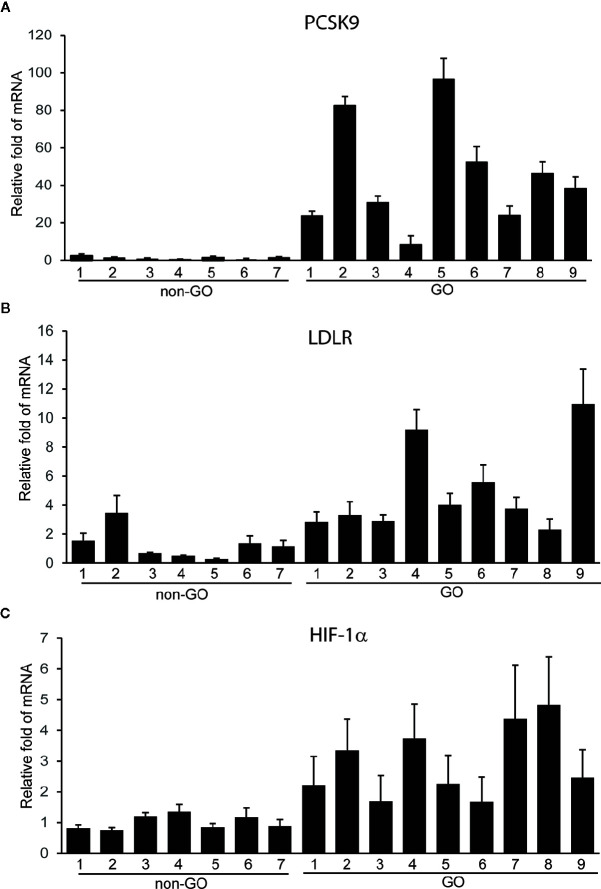
Expression of proprotein convertase subtilisin/kexin type 9 (PCSK9), low density lipoprotein receptor (LDLR), and hypoxia-inducible factor 1a (HIF-1a) mRNAs in Graves’ orbitopathy (GO) and non-GO orbital tissues. The RNA extracted from GO (n=9) and non-GO (n=7) orbital tissues was reverse-transcribed by real-time PCR and quantified. Experiments were performed in triplicate for each donor. The results showed elevated transcript levels of PCSK9 **(A)**, LDLR **(B)**, and HIF-1a **(C)** in GO tissues than in non-GO tissues. Data in the column indicate the mean ± SD fold elevation relative to the control.

To investigate its potential role in GO, we measured the expression of PCSK9 in orbital tissues taken from GO and non-GO subjects. The RT-PCR results showed that PCSK9 transcript levels were greater in GO tissues (n=9) than in non-GO tissues (n=7) (**Figure 1A**). Additionally, the mRNA levels of LDLR and hypoxia-inducible factor 1α (HIF-1α) were higher in GO tissues (n=9) than in non-GO tissues (n=7) (**Figures 1B, C**).

### IL-1β Induces PCSK9 and LDLR in Orbital Fibroblasts

**Figure 2:**
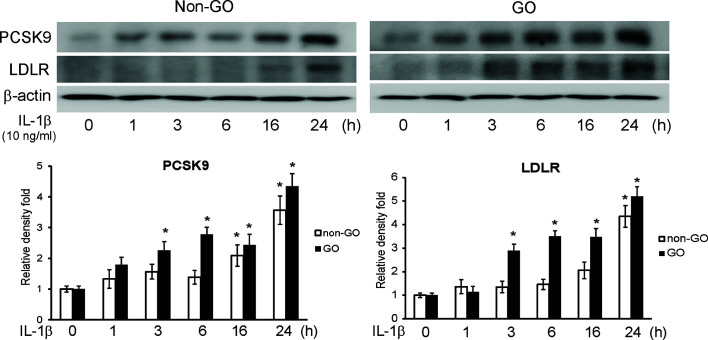
Western blot of proprotein convertase subtilisin/kexin type 9 (PCSK9) and low density lipoprotein receptor (LDLR) after interleukin-1β (IL-1β) treatment. Confluent orbital fibroblasts obtained from Graves’ orbitopathy (GO) (n=3) and non-GO subjects (n=3) were treated with 10 ng/ml of IL-1β for increasing lengths of time (0–24 h). Western blot analyses were performed to investigate the levels of PCSK9 and LDLR. The treatment with IL-1β increased the levels of PCSK9 and LDLR in both GO and non-GO tissues in a time-dependent manner. Representative gel images are shown. Data in the columns indicate the mean density ratio ± SD, normalized to the level of *β*-actin in the same sample (*p < 0.05 vs. 0 h in each group).

We challenged GO and non-GO orbital fibroblasts with IL-1β, a key mediator in GO (18, 22), for 1, 3, 6, 16, and 24 h. The western blot results showed that IL-1β led the GO and non-GO fibroblasts to increase PCSK9 and LDLR expression in a time-dependent manner (**Figure 2**). The increase in PCSK9 and LDLR was more prominent in GO than in non-GO fibroblasts.

### Silencing PCSK9 Suppresses IL-1β-Induced Expression of Pro-Inflammatory Mediators

**Figure 3:**
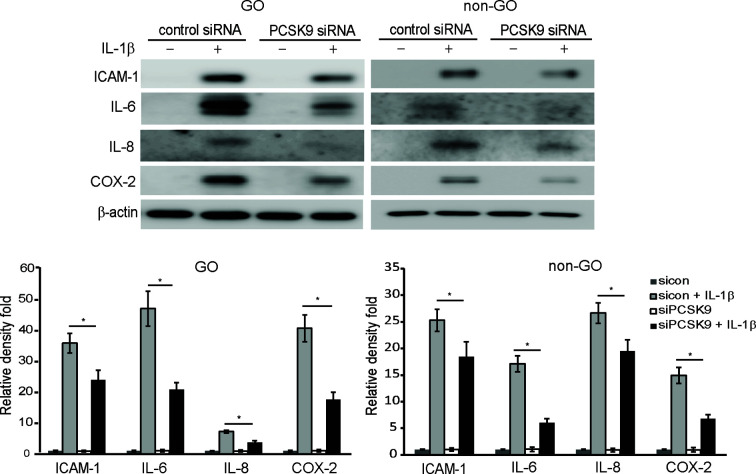
Effect of silencing proprotein convertase subtilisin/kexin type 9 (PCSK9) on the expression of pro-inflammatory cytokines protein. Confluent fibroblasts obtained from Graves’ orbitopathy (GO) patients (n=3) were treated with either control siRNA or PCSK9 siRNA (50 nM, 24 h). Then, they were challenged with 10 ng/ml of interleukin-1β (IL-1β) and compared to non-IL-1β-treated counterparts. Western blot analyses were conducted to compare the levels of pro-inflammatory cytokines, intercellular adhesion molecule 1 (ICAM-1), IL-6, IL-8, and cyclooxygenase-2 (COX-2). The same experiment was repeated with fibroblasts obtained from non-GO subjects (n=3). Representative gel images are shown. The mean density ratio ± SD from fibroblasts were normalized to the level of *β*-actin in the same sample (*p < 0.05 between sicon + IL-1β and siPCSK9 + IL-1β; sicon, control siRNA; siPCSK9, PCSK9 siRNA).

The western blot results showed that when orbital fibroblasts were transfected with PCSK9 siRNA, the production of pro-inflammatory mediators, ICAM-1, IL-6, IL-8, and cyclooxygenase-2 (COX-2), in response to the challenge with IL-1β was significantly suppressed in GO and non-GO fibroblasts (**Figure 3**).

### Silencing PCSK9 Reduces Activation of Signaling Molecules

**Figure 4:**
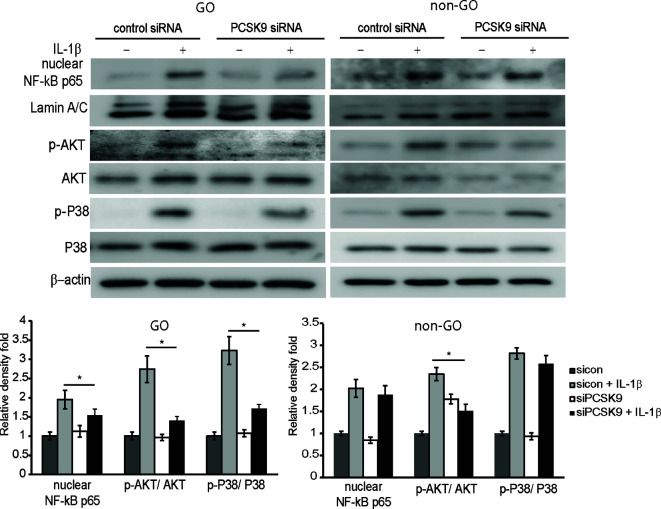
Effect of silencing proprotein convertase subtilisin/kexin type 9 (PCSK9) on the activation of signal molecules by interleukin-1β (IL-1β) treatment. Confluent orbital fibroblasts obtained from Graves’ orbitopathy (GO) patients (n=3) were treated with or without 10 ng/ml of IL-1β after transfection with control siRNA or PCSK9 siRNA (50 nM, 24 h). Treatment with IL-1β (10 ng/ml, 60 min) resulted in an increase in the levels of nuclear NF-κB p65 and phosphorylated forms of Akt and p38. The treatment with PCSK9 siRNA in GO cells significantly blunted the increases in the transcription factors. However, in fibroblasts from non-GO subjects (n=3), the PCSK9 inhibition only suppressed the phosphorylated Akt. Representative gel images are shown. Data in the columns indicate the mean density ratio ± SD of the bands obtained from the GO patients, normalized to the level of *β*-actin in the same sample (*p < 0.05 between sicon + IL-1β and siPCSK9 + IL-1β).

As shown in **Figure 4**, the IL-1β-treatment (10 ng/ml for 60 min) increased levels of nuclear NF-κB p65 and phosphorylated (p-) forms of Akt and p38 in GO and non-GO fibroblasts in western blot analyses. In GO fibroblasts, PCSK9 interference with siRNA reduced IL-1β-stimulated expression of nuclear NF-κB p65, p-Akt, and p-p38. In non-GO fibroblasts, the PCSK9 inhibition suppressed IL-1β-stimulated expression of p-Akt, but not NF-κB p65 and p-p38.

### Silencing PCSK9 Inhibits Proliferation of GO Fibroblasts

The enhanced proliferative capacity of GO fibroblasts at baseline and in response to certain cytokines may play a role in the pathogenesis of GO (23). According to the MTT assay results, the proliferation of GO fibroblasts slowed down 48 h after being transfected with PCSK9 siRNA compared to control siRNA-treated group (**Supplementary Figure 1**).

### PCSK9 Inhibition Suppresses Adipocyte Differentiation and Oxidative Stress-Related Protein Production in GO Fibroblasts

**Figure 5:**
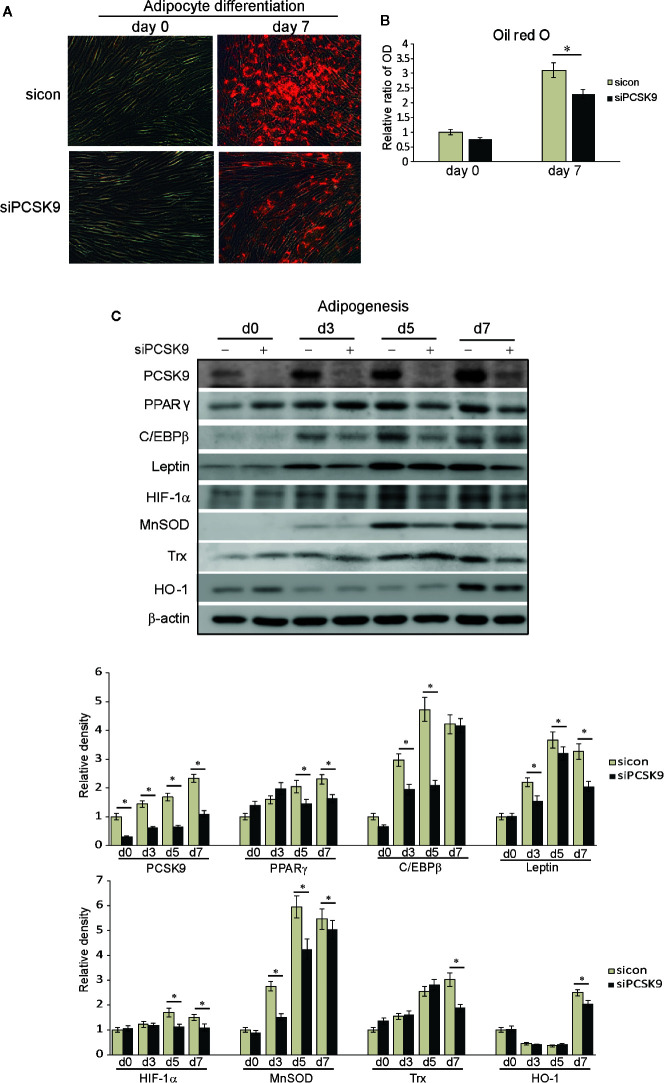
Proprotein convertase subtilisin/kexin type 9 (PCSK9) siRNA suppresses adipogenesis and oxidative stress in Graves’ orbitopathy (GO) fibroblasts. Orbital fibroblasts from GO (n=3) patients were cultured in adipogenic medium to induce differentiation into adipocytes for 7 days. **(A)** Oil red O staining showed treatment with PCSK9 siRNA (50 nM, 24 h) attenuated adipogenesis. **(B)** Quantification by measurements of optical density of cell lysates at 490nm echoed the histochemical results. The results are presented as the mean optical ratio ± SD (*p < 0.05 between sicon and siPCSK9). **(C)** Western blot analyses showed that throughout the 7-day period of adipogenesis, the cell lysates of fibroblasts collected at different time points showed a gradual increase in production of PCSK9, which was markedly decreased when PCSK9 siRNA (50 nM, 24 h) was treated. The levels of adipogenic transcription factors, peroxisome proliferator-activated receptor gamma (PPARγ) and CCAAT/enhancer-binding protein β (C/EBPβ), were substantially curtailed in fibroblasts transfected with PCSK9 siRNA. The levels of mature adipocyte marker, leptin, were also significantly reduced in the PCSK9 siRNA-treated group. The levels of antioxidants, manganese superoxide dismutase (MnSOD), thioredoxin (Trx), and heme oxygenase-1 (HO-1), and oxidative stress-related protein, hypoxia-inducible factor 1α (HIF-1α) were significantly decreased in the PCSK9 siRNA-transfected group. Data in the columns indicate the mean density ratio ± SD, normalized to the level of *β*-actin in the same sample, and representative gel images are shown (*p < 0.05 between sicon and siPCSK9 on days 0, 3, 5, and 7 of adipogenesis).

The transfection of differentiating fibroblasts with PCSK9 siRNA attenuated adipogenesis on day 7 according to the Oil Red O staining (**Figure 5A**). When quantified by measuring optical density of Oil Red O-stained cell lysates at 490 nm, the same pattern was identified (**Figure 5B**). Throughout adipogenic differentiation, the PCSK9 levels gradually increased (**Figure 5C**), but PCSK9 siRNA substantially diminished levels of adipogenic transcription factors, PPARγ and CCAAT/enhancer-binding protein β (C/EBPβ), and mature adipocyte marker, leptin. In addition, silencing PCSK9 markedly decreased the levels of oxidative stress-related protein, HIF-1α, and antioxidant proteins, manganese superoxide dismutase (MnSOD), thioredoxin (Trx), and heme oxygenase-1 (HO-1) (**Figure 5C**).

### Plasma PCSK9 Protein Levels Are Elevated in GO and Plasma PCSK9 Concentrations Show Positive Correlations With Plasma TBII Levels and CAS

**Figure 6:**
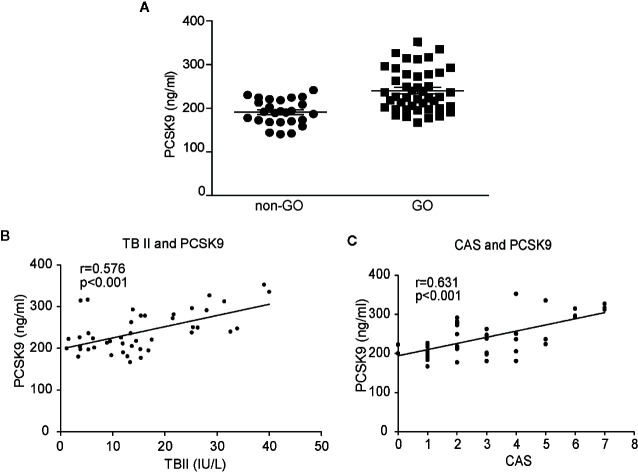
Comparison of plasma levels of proprotein convertase subtilisin/kexin type 9 (PCSK9) between Graves’ orbitopathy (GO) patients and non-GO subjects, and correlation analyses between plasma levels of PCSK9, thyrotropin-binding inhibitor immunoglobulin and clinical activity score (CAS). The plasma levels of PCSK9 were measured in GO patients and non-GO subjects using ELISA. The samples were assayed in triplicate. **(A)** The mean PCSK9 plasma level was significantly higher in the GO patients (n=44, 239.97 ± 48.20 ng/ml) than the healthy subjects (n=26, 190.83 ± 28.77 ng/ml; *p* < 0.01). A single dot represents the value obtained from a single donor. The results of Spearman’s rank correlation test between plasma levels of PCSK9 (GO patients, n=44) and **(B)** plasma levels of thyrotropin binding inhibitory immunoglobulin (TBII) (GO patients, n=44) or **(C)** clinical activity score (CAS) (GO patients, n=44) are shown. The plasma PCSK9 concentrations showed significant associations with the plasma TBII levels (r = 0.576, *p* < 0.001) and CAS (r = 0.631, *p* < 0.001).

The plasma PCSK9 levels were measured in GO and healthy subjects by ELISA (**Figure 6A**). The mean plasma PCSK9 level was significantly higher in GO patients (n=44, 239.97 ± 48.20 ng/ml) than in healthy subjects (n=26, 190.83 ± 28.77 ng/ml; *p* < 0.01). Additionally, the mean plasma PCSK9 level was significantly higher in patients with active GO (n=22, 256.46 ± 53.49 ng/ml) than in patients with inactive GO (n=22, 223.48 ± 36.42 ng/ml; *p*=0.01). The plasma PCSK9 level was correlated with TBII (*r* = 0.576, *p* < 0.001, n = 44; **Figure 6B**) and CAS (*r* = 0.631, *p* < 0.001, n = 44; **Figure 6C**).

## Discussion

In this study, we examined the role of PCSK9 in GO pathogenesis. GO tissues showed increased expression of *PCSK9*, *LDLR*, and *HIF-1α*. The IL-1β challenge and adipogenic stimulation led to the increase of PCSK9 and LDLR. PCSK9 silencing with siRNA significantly decreased pro-inflammatory cytokines production, oxidative stress-related proteins, adipogenic transcription factors, and adipocyte differentiation. Importantly, the plasma level of PCSK9 was elevated in GO patients when compared to the non-GO subjects. It also showed a positive correlation with CAS, a measure of GO activity as well as TBII, a predictor of GO severity (17, 24).

Numerous studies have attempted to clarify the pro-inflammatory roles of PCSK9 in a variety of disorders including atherosclerosis, sepsis, psoriasis, steatosis, and myocardial ischemia (12–14, 25–30). For example, Tang et al. suggested an atherogenic role of PCSK9 as the suppression of PCSK9 expression in *apoE* null mice by means of small hairpin RNA decreased expression of TNF-α, IL-1β, monocyte chemotactic protein 1, Toll-like receptor 4, and NF-κB and reduced macrophage infiltration in the atherosclerotic plaques (26). In other *in vivo* and *in vitro* studies, the PCSK9 inhibition diminished pro-inflammatory cytokines production and macrophage accumulation by inhibiting NF-κB activation (12, 27, 30). In the context of GO, however, there have been no studies on the role of PCSK9. To the best of our knowledge, this study is the first of its kind to identify the pro-inflammatory properties of PCSK9 in GO. Based on our results, silencing PCSK9 ameliorated inflammation by modulating NF-κB pathway. Furthermore, the PCSK9 level was higher in GO tissues compared to the control at baseline, and IL-1β, a key mediator in GO inflammation (18, 22), increased PCSK9 levels more prominently in GO fibroblasts than in the control. Our study suggests that PCSK9 may serve as a therapeutic target for GO inflammation.

Mounting evidence has shown the anti-adipogenic effects of PCSK9 depletion (13, 31–33). Currently, PPARγ and C/EBPβ are believed to be responsible for terminal differentiation of fibroblasts into adipocytes (34). The PPARγ activation leads to the expression of adipogenic markers including leptin and fatty acid synthase (FAS) (35). Upstream of PPARγ, molecules such as E2F1 is thought to be involved (36). Recently, a study on rat models with alcohol-induced steatosis showed that the treatment with alirocumab, a human PCSK9 monoclonal antibody, attenuated expression of FAS and alleviated alcohol-induced lipid accumulation. Moreover, PCSK9 inhibition reduced mRNA expression of E2F1 as well as sterol regulatory element-binding protein (SREBP)-1 and SREBP-2 (13). SREBP-1 and SREBP-2 have been found to regulate cholesterol- and fatty acid metabolism-related genes (37). Ruscica et al. also showed that, in 201 patients with suspected nonalcoholic steatosis, hepatic PCSK9 mRNA levels were correlated with hepatic SREBP-1 and FAS expression (31). These results are consistent with the those of our own study; the PCSK9 inhibition attenuated adipogenesis as identified by Oil Red O and blunted the expression of PPARγ, C/EBPβ, and leptin. Furthermore, the PCSK9 expression gradually increased throughout the adipogenic differentiation. In this study, we present the evidence for the adipogenic role of PCSK9 in GO. However, whether PCSK9 directly modulates PPARγ or C/EBPβ activity needs to be further investigated. Furthermore, as PPARγ agonists have recently been suggested to modulate helper T cell-related chemokine production in GO (38), further studies are needed to examine if PCSK9 intervention modulates PPARγ-mediated inflammation.

Adipogenesis is thought to be closely associated with oxidative stress (39), and both are found by our study to be significantly suppressed by the PCSK9 inhibition. Multiple studies have proven the anti-oxidative effects of PCSK9 suppression in several disorders including atherosclerosis and myocardial infarction (14, 40). Locally produced reactive oxygen species (ROS) leads to the oxidation of LDL and contributes to atherogenesis (41). Transfection of endothelial cells and vascular smooth muscle cells with PCSK9 siRNA substantially decreased the production of ROS (40). In several *in vitro* studies, PCSK9 inhibition reduced ROS generation, while PCSK9 overexpression produced the opposite results (14, 42–44). In an *in vivo* study, PCSK9 knockout mice expressed significantly less NADPH oxidase and subsequently less ROS in aorta (40). These results are in line with those of our study. As antioxidants, Trx, MnSOD, and HO-1 are induced by oxidative stress and protect tissues from oxidative injuries (45–47). Under hypoxia, HIF-1α is activated to increase the expression of genes involved in adipogenesis and tissue remodeling in GO (39). Our results demonstrated that PCSK9 inhibition blunted the level of oxidative stress-related proteins which was induced by adipocyte differentiation. Previously, we have found that quercetin inhibits cigarette smoke extract-induced adipogenesis in GO fibroblasts by reducing ROS (48). Additionally, we have reported several molecules with anti-oxidative properties such as resveratrol, caffeine, and curcumin suppress adipogenesis in GO fibroblasts (49–51). Given that oxidative stress contributes to the proliferation of orbital fibroblasts (52), the impeded proliferation of GO fibroblasts by PCSK9 inhibition demonstrated in this study may be attributed to the anti-oxidative property of the PCSK9 inhibitor.

The changes in the level of phosphorylated forms of transcription factors upon the transfection of PCSK9 siRNA in GO fibroblasts indicate that a complex network of signaling pathways may exist. In this study, the PCSK9 inhibition decreased the activation of Akt and attenuated adipogenesis in GO fibroblasts. Our study with siRNA transfection is limited by the lack of mRNA data, which would have provided additional insights to its effect at transcriptional or translational level. However, like other studies that have employed similar methods (53, 54), we believe that we have shown a concrete evidence with western blot and ELISA that PCSK9, regardless of the mechanism with which its level is modified, affects inflammation and adipogenesis in GO. In line with our study, Kumar et al. have previously reported that an autoantibody against TSH receptor stimulated the phosphoinositide 3-kinase (PI3K)/Akt pathway and induced adipogenesis of orbital preadipocytes in GO (55). They asserted that this pathway triggered the terminal stages of adipogenesis. Another recent study by our group has presented that an Akt inhibitor suppressed adipogenesis in GO orbital fibroblasts (56). Another downstream effector of PI3K/Akt pathway, Forkhead box O (FOXOs), has also been demonstrated to repress excessive adipogenesis and hyaluronan overproduction in GO fibroblasts (57). As multiple studies, including our own, continue to highlight the importance of the PI3K/Akt pathway in the GO pathogenesis, further studies are necessary to identify the interaction between the signaling molecules as well as PCSK9.

Finally, we show that plasma PCSK9 levels were significantly higher in active GO patients than inactive GO patients as well as healthy subjects. These results, along with the higher PCSK9 mRNA levels in GO tissues, strongly suggest the involvement of PCSK9 in GO pathogenesis. Moreover, the plasma PCSK9 level revealed a strong correlation with plasma TBII level as well as CAS. Its role in many other inflammatory and autoimmune diseases such as systemic lupus erythematosus, rheumatoid arthritis, and type 1 diabetes demonstrates its complex biological activity (58–60). Identifying new biomarkers and therapeutic targets such as PCSK9 can further our knowledge of these disorders and lead to the development of effective treatments.

In conclusion, we demonstrated that PCSK9 inhibition countered pro-inflammatory cytokines production, oxidative stress-related proteins, adipogenic transcription factors, and adipocyte formation in GO fibroblasts. The PCSK9 level was increased during the IL-1β challenge and adipogenic stimulation. The plasma PCSK9 level was elevated in GO patients and positively correlated with clinical inflammation and thyrotropin receptor antibody titer, indicating that PCSK9 is a potential biomarker for diagnosis and prognosis of GO. Further studies are needed to establish the response to PCSK9 inhibitors *in vivo* and explore the use of the inhibitor as an effective therapeutic strategy for GO.

## Data Availability Statement

The raw data supporting the conclusions of this article will be made available by the authors, without undue reservation.

## Ethics Statements

The studies involving human participants were reviewed and approved by Institutional Review Board of Severance Hospital. The patients/participants provided their written informed consent to participate in this study.

## Author Contributions

GL drafted the manuscript; JiK acquired and interpreted the data. JL, JaK, and EL revised the work. JY designed the study and revised the work. All authors contributed to the article and approved the submitted version.

## Funding

This study was supported by a faculty research grant of Yonsei University College of Medicine (6-2020-0093).

## Conflict of Interest

The authors declare that the research was conducted in the absence of any commercial or financial relationships that could be construed as a potential conflict of interest.

## Supplementary Material

Supplementary Figure 1 siRNA-mediated knockdown of PCSK9 in GO and non-GO fibroblasts. GO (n=3) and non-GO (n=3) orbital fibroblasts were transfected with PCSK9 siRNA (50, 100 nM) for 24 h. As shown in western blot results, the level of PCSK9 was significantly decreased following RNA interference. *β* **-**actin was included as a loading control. NC, normal control.

Supplementary Figure 2 Effect of PCSK9 siRNA on viability of GO fibroblasts. Orbital fibroblasts of GO patients (n=3) were seeded in 24-well culture plates, 1 × 10^5^ cells per well. PCSK9 siRNA (50 nM) were applied to wells for 10, 24, and 48 h and MTT assay was conducted to test cell viability. Results are presented as mean ± SD. Assays were carried out in triplicate and repeated at least three times. The proliferation of GO fibroblasts was impeded in PCSK9 siRNA-treated fibroblasts compared to the control.
