# Supplementary figures and images for: Role of Proprotein Convertase Subtilisin/Kexin Type 9 in the Pathogenesis of Graves’ Orbitopathy in Orbital Fibroblasts
Source: Front Endocrinol (Lausanne). 2021 Jan 8;11:607144. doi: 10.3389/fendo.2020.607144 (PMC7821242; doi:10.3389/fendo.2020.607144)

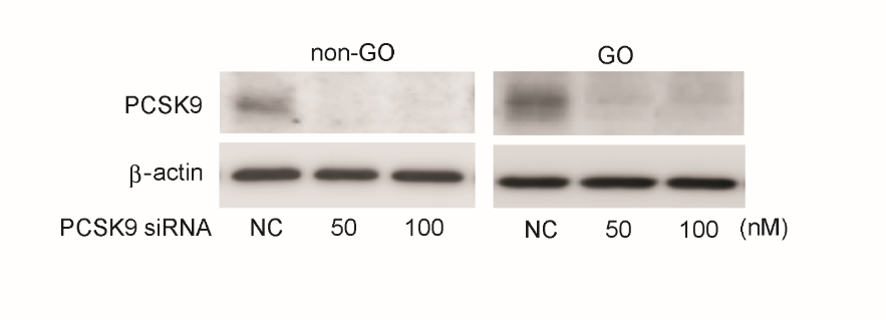

Supplement: Supplementary Figure 1 — siRNA-mediated knockdown of PCSK9 in GO and non-GO fibroblasts. GO (n=3) and non-GO (n=3) orbital fibroblasts were transfected with PCSK9 siRNA (50, 100 nM) for 24 h. As shown in western blot results, the level of PCSK9 was significantly decreased following RNA interference. β -actin was included as a loading control. NC, normal control. [file Image_1.tif]

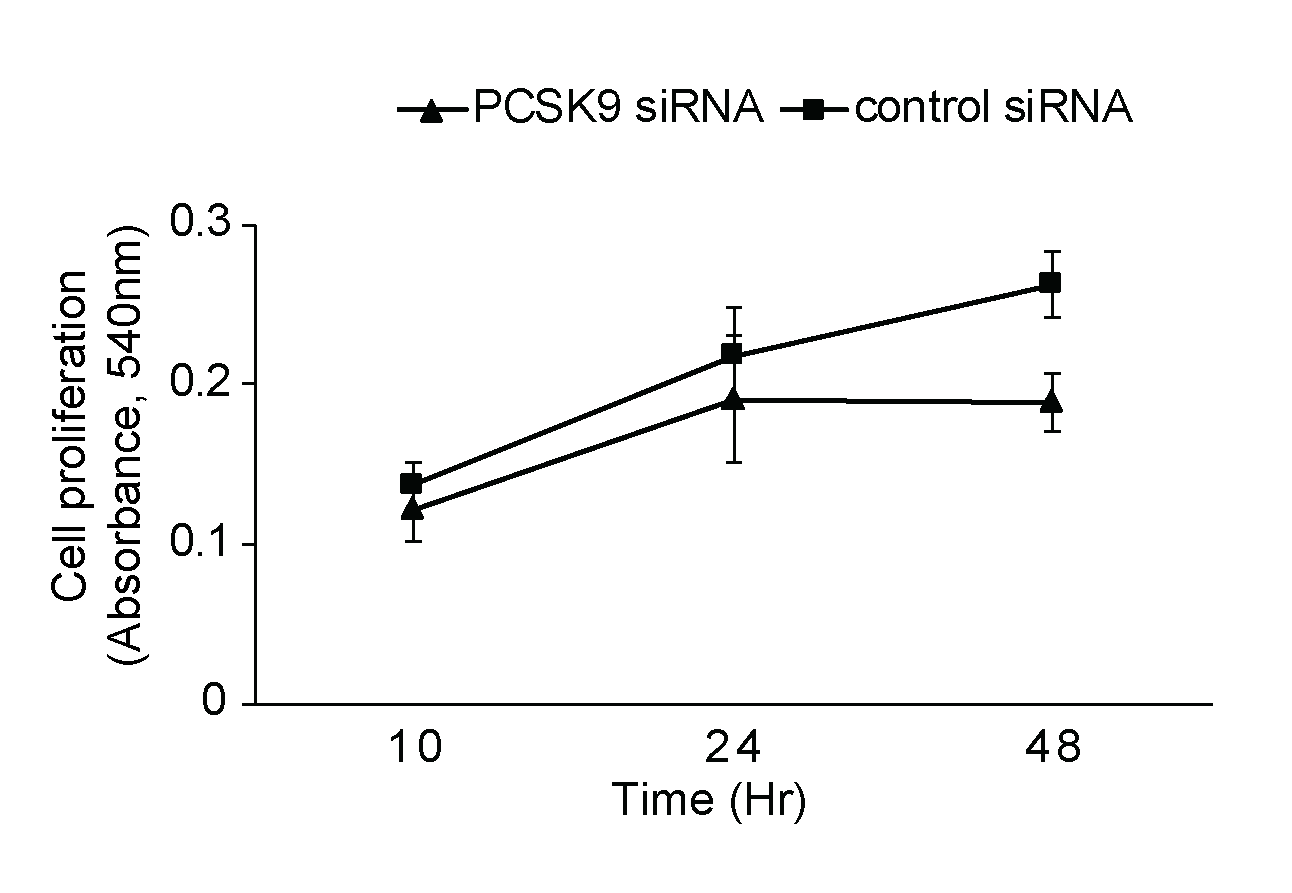

Supplement: Supplementary Figure 2 — Effect of PCSK9 siRNA on viability of GO fibroblasts. Orbital fibroblasts of GO patients (n=3) were seeded in 24-well culture plates, 1 × 10^5^ cells per well. PCSK9 siRNA (50 nM) were applied to wells for 10, 24, and 48 h and MTT assay was conducted to test cell viability. Results are presented as mean ± SD. Assays were carried out in triplicate and repeated at least three times. The proliferation of GO fibroblasts was impeded in PCSK9 siRNA-treated fibroblasts compared to the control. [file Image_2.tif]
