# Supplementary material for: Role of Proprotein Convertase Subtilisin/Kexin Type 9 in the Pathogenesis of Graves’ Orbitopathy in Orbital Fibroblasts
Source: Front Endocrinol (Lausanne). 2021 Jan 8;11:607144. doi: 10.3389/fendo.2020.607144 (PMC7821242; doi:10.3389/fendo.2020.607144)
Supplement: Supplementary file 3 [file Table_1.docx]

**SUPPLEMENTARY TABLE S1.** LIST OF REAGENTS

| *Antibody** | *Manufacturer* | *Catalogue number* | *Species* |
| --- | --- | --- | --- |
| PCSK9 | Santa Cruz Biotechnology | sc-515082 | Human |
| LDLR | Santa Cruz Biotechnology | sc-373830 | Human |
| β-actin | Santa Cruz Biotechnology | sc-47778 | Human, Mouse, Rat |
| ICAM-1 | Cell Signaling Technology | 4915 | Human |
| IL-6 | Novus Biologicals | NB600-1131 | Human, Mouse, Rat |
| IL-8 | Abcam | Ab7747 | Human |
| COX-2 | Cell Signaling Technology | 4842 | Human, Mouse, Rat |
| P65 NF-κB | Santa Cruz Biotechnology | 4764 | Human, Mouse, Rat |
| Lamin A/C | Santa Cruz Biotechnology | Sc-376248 | Human, Mouse, Rat |
| p-Akt | Cell Signaling Technology | 9271 | Human, Mouse, Rat |
| t-Akt | Cell Signaling Technology | 9272 | Human, Mouse, Rat |
| p-p38 | Cell Signaling Technology | 9211 | Human, Mouse, Rat |
| t-p38 | Cell Signaling Technology | 9212 | Human, Mouse, Rat |
| PPARγ | Santa Cruz Biotechnology | sc-7273 | Human, Mouse, Rat |
| C/EBPβ | Santa Cruz Biotechnology | sc-7962 | Human, Mouse, Rat |
| Leptin | Santa Cruz Biotechnology | sc-48408 | Human |
| HIF-1α | Santa Cruz Biotechnology | sc-53546 | Human, Mouse, Rat |
| MnSOD | Santa Cruz Biotechnology | sc-30080 | Human, Mouse, Rat |
| Trx | Santa Cruz Biotechnology | sc-20146 | Human |
| HO-1 | Assay design | OSA-110 | Human, Mouse, Rat, Bovine, Dog |

Abbreviations: PCSK9, proprotein convertase subtilisin/kexin type 9; LDLR, low density lipoprotein receptor; ICAM-1, anti-intercellular adhesion molecule 1; COX-2, cyclooxygenase-2; NF-κB, nuclear factor kappa-light-chain-enhancer of activated B; PPARγ, peroxisome proliferator activator gamma; C/EBP, CCAAT-enhancer-binding protein; HIF-1, hypoxia-inducible factor-1; MnSOD, manganese superoxide dismutase; Trx, thioredoxin; HO-1, heme oxygenase-1.

*The antibodies are listed in the order of appearance.
