# Supplementary material for: Role of Proprotein Convertase Subtilisin/Kexin Type 9 in the Pathogenesis of Graves’ Orbitopathy in Orbital Fibroblasts
Source: Front Endocrinol (Lausanne). 2021 Jan 8;11:607144. doi: 10.3389/fendo.2020.607144 (PMC7821242; doi:10.3389/fendo.2020.607144)
Supplement: Supplementary file 4 [file Table_2.docx]

SUPPLEMENTARY TABLE S2. PRIMER SEQUENCE FOR RT-PCR

| *Primer* | *Direction* | *Sequence* |
| --- | --- | --- |
| GAPDH | Forward | 5´-TGC TGT AGC CAA ATT CGT TG-3´ |
|  | Reverse | 5´-CAC CCA CTC CTC CAC CTT T-3´ |
| PCSK9 | Forward | 5´-TGG AAC TCA CTC ACT CTG GG-3´ |
|  | Reverse | 5´-AAG AAT CCT GCC TCC TTG GT-3´ |
| LDLR | Forward | 5´-GAC GTG GCG TGA ACA TCT G-3´ |
|  | Reverse | 5´-CTG GCA GGC AAT GCT TTG G-3´ |
| HIF-1α | Forward | 5´-TTC ACC TGA GCC TAA TAG TCC -3´ |
|  | Reverse | 5´-CAA GTC TAA ATC TGT GTC CTG-3´ |

Abbreviations: GAPDH, glyceraldehyde-3-phosphate dehydrogenase; PCSK9, proprotein convertase subtilisin/kexin type 9; LDLR, low density lipoprotein receptor; HIF-1, hypoxia-inducible factor-1.
